# Effect of the Rare Earth Element Cerium on the Corrosion Resistance of Steel for an Offshore Platform in a Simulated Marine Atmospheric Environment

**DOI:** 10.3390/ma18112443

**Published:** 2025-05-23

**Authors:** Yanjie Wu, Ruifeng Dong, Zhipeng Mu, Jing Guo, Xiong Yang, Peiying Zhou

**Affiliations:** 1 School of Materials Science and Engineering, Inner Mongolia University of Technology, Hohhot 010051, China; 15391041563@163.com (Y.W.); mzp17702449605@163.com (Z.M.);; 2 Engineering Research Center of Rare Earth Metal Materials, Inner Mongolia University of Technology, Hohhot 010051, China; 3 Fushun Special Steel Co., Ltd., Fushun 113001, China; 4 The Rare Earth Steel Plates Plant of Baotou Iron and Steel Co., Ltd., Baotou 014010, China; 5 Technical Center of Inner Mongolia Baotou Steel Union Co., Ltd., Baotou 014010, China

**Keywords:** rare earth elements, rust layer, corrosion behavior, simulation calculations

## Abstract

This study investigated the corrosion behavior and mechanism of offshore platform steel in a simulated marine atmospheric environment using electrochemical analysis, phase analysis, and rust layer characterization. The findings demonstrate that the addition of trace amounts of the rare earth element Ce significantly enhances the steel’s corrosion resistance in the marine environment and effectively reduces the corrosion rate. The addition of Ce promotes the enrichment of Cr in the inner rust layer and forms a dense protective rust layer, thereby preventing the rust layer from falling off, as well as hindering the penetration of oxygen ions. Phase analysis and electrochemical studies further confirmed that the addition of rare earth Ce optimized the structure of the rust layer, improved the matrix protection effect, and enhanced the corrosion resistance of the steel. The crystal structure of the rust layer and the stability between the matrix were simulated by first principles. The results show that the addition of rare earth enhances the bonding force and compactness of the steel matrix and the rust layer, thereby improving corrosion resistance.

## 1. Introduction

Due to the huge structure of offshore platforms and the fact that most of them are located underwater, corrosion detection and monitoring are difficult, which may lead to corrosion problems that cannot be found and treated in time, increasing the complexity of corrosion protection. In response to these environmental challenges, offshore platform steels are usually protected by protective measures such as coating protection, cathodic protection, and corrosion-resistant materials, and are regularly tested and maintained to ensure their safe long-term operation. However, these means of protection are often costly and difficult to construct, generate hazardous waste, increase the environmental burden, or need to be replaced regularly, increasing maintenance costs. Therefore, it is very important to improve the corrosion resistance of steel [1,2]. The corrosion protection of offshore platform steel needs to comprehensively consider the environment, material properties, and economic costs to ensure the safety and long-term use of the platform. In recent years, rare earth elements have been used in microalloyed steels as modifiers and key microalloying elements due to their active structure and unique chemical activity [3,4,5]. Studies have shown that rare earth elements can purify molten steel [6], reducing the content of inclusions and improving the morphology of inclusions [6,7,8], affecting the corrosion behavior of steel. The modification of rare earth elements can not only reduce the size and density of inclusions but also reduce the initiation site of pitting corrosion and increase the pitting potential [7,9]. At the same time, Liu et al. [10] found that rare earth-modified inclusions preferentially dissolve in the early stage of corrosion, thereby protecting the surrounding matrix. For the preferential dissolution behavior of RE-O-S inclusions, Wei et al. [11] proposed a theoretical explanation through first-principles calculations. However, the effect of rare earth on the corrosion of steel is the result of many factors. Inclusions often affect the initial corrosion process [8], while the corrosion products and compactness of the rust layer have an important influence on long-term corrosion [10]. In previous studies, most rare earth additions were high, which could easily lead to clogging of the casting nozzle during the continuous casting process, which in turn leads to casting interruption, molten steel return, reduced tundish life, and increased production costs. The generation of a blockage may also cause problems, such as sticking breakout and surface quality defects in the slab. If the blockage falls off into the molten steel, it may affect the purity of the molten steel and produce large size inclusions, which will reduce the quality of the slab [12]. Therefore, it is crucial to study the mechanism of influence for trace rare earth elements on the corrosion behavior of steel used in offshore platforms. This not only enhances the corrosion resistance of rare earth elements but also helps avoid negative impacts on production efficiency and costs, which is a significant factor in promoting the industrial application of rare earth elements. In this study, SEM, XRD, an electrochemical workstation, and first-principles calculations were used to investigate the effect of rare earth elements on the corrosion performance of offshore platform steel. Through electrochemical impedance spectroscopy (EIS) and potentiodynamic polarization methods, the corrosion behavior of different experimental steels in chloride-containing solutions was analyzed. The electron probe micro-analyzer (EPMA) was employed to observe the segregation of alloy elements between the rust layer and the steel substrate. Compared to existing research on corrosion resistance, the innovation of this study lies in systematically elucidating both the distribution of cerium in the microstructure and its role in promoting the formation of rust layers through experimental work and first-principles simulations. This finding provides new insights for the future development of steel under harsh marine conditions.

## 2. Experimental Materials and Methods

### 2.1. Experimental Materials

Three types of steel samples were used in this experiment, and their chemical compositions are listed in Table 1. Sample ^#^17 did not contain rare earth elements, while sample ^#^1 was alloyed with 6 ppm of rare earth elements, and sample ^#^3 was alloyed with 21 ppm of rare earth elements.

### 2.2. Experimental Methods

The samples were machined into dimensions of 20 × 30 × 3 mm, using a wire cutting machine, for the weight loss tests, rust layer composition analysis, microstructure and macrostructure observation, and electrochemical analysis. To facilitate suspension, all corrosion samples had 1.5 mm holes drilled 2 mm from the edge. The surface roughness of the samples was processed to Ra 0.8. After grinding, the samples were cleaned using an ultrasonic cleaner, blow-dried, weighed on a high-precision electronic balance, and stored in a drying oven for later use.

## 3. Structural Characterization and Corrosion Resistance Testing

The experiment simulated a marine atmospheric corrosion environment following the GB-T10125-2012 [13] neutral salt spray test standard. The samples were placed in a salt spray chamber at 25 °C, using a hydraulic atomization spray mode for continuous spraying. The samples were suspended in the chamber, with the corrosion solution consisting of 3.5% NaCl. The corrosion periods were set to 7 days, 14 days, 21 days, and 28 days. After each period, the samples were rinsed with water, blow-dried, and the macroscopic appearance of the rust layer was recorded.

### Corrosion Treatment

(1) Surface Morphology Observation: A digital camera was used to capture the macroscopic appearance of the rust layer. Selected samples were cold-mounted and gold-sprayed, then the cross-sections of the rust layers were observed using an S-3400N SEM (Hitachi, Tokyo, Japan). A Zeiss LSM 900 confocal laser scanning microscope (CarlZeiss AG, Oberkochen, Germany) was used to observe the depth and smoothness of the corrosion pits on the derusted surfaces after the first and fourth cycles.
(2) Corrosion Weight Loss Measurement: Following the GB/T16545-2015 [14] standard, a rust removal solution was prepared using 500 mL HCl (ρ = 1.19 g/mL), 3.5 g hexamethylenetetramine, and distilled water to make up 1000 mL. The samples were immersed in the rust removal solution and treated with ultrasonic cleaning. After the rust layer was removed, the samples were rinsed with distilled water and alcohol, then blow-dried. Once dry, the samples were weighed using a high-precision electronic balance, and the weight loss was recorded. The corrosion rate of the three test steels was calculated using the weight loss method, and corrosion rate curves for each period were plotted.
(3) Rust Layer Phase Analysis: The rust layer was scraped from the samples and ground to a micron-sized powder using a mortar. The powder was then sieved through a 2000-mesh screen. A Bruker D8 ADVANCE multifunctional X-ray diffractometer (Rigaku Corporation, Tokyo, Japan) was used to analyze the phase structure, with a scanning range of 10° to 80°, a scanning rate of 5°/min, a voltage set to 40 kV, and a current of 30 mA. Data analysis was performed using Jade 9 software.
(4) Electrochemical Impedance Spectroscopy (EIS) Testing (Zahner-Elektrik GmbH & Co. KG, Kronach, Germany): EIS tests were conducted using a Zennium Pro electrochemical workstation with a three-electrode system. The working electrode was the rusted sample, the reference electrode was a saturated calomel electrode (SCE), and the counter electrode was a platinum sheet. The electrolyte used was a 3.5 wt.% NaCl solution. To ensure sample stability and consistency with the experimental environment, the samples were immersed in the corrosion solution for approximately 20 min before measuring the open circuit potential, with the test lasting 1000 s. The frequency range was 10^−^^2^ Hz to 10^5^ Hz, with a sinusoidal excitation signal amplitude of 10 mV.
(5) Simulation Calculations: The VASP software (Version 4.5), based on density functional theory (DFT), was used to perform stability calculations for the system, describing the ground-state properties of the material, including its energy, lattice structure, and electronic structure. The adhesion work was calculated to evaluate the bonding stability between the rust layer and the substrate, which allowed for an assessment of the system’s energy stability.

## 4. Results and Discussion

### 4.1. Rust Layer Morphology Analysis

#### 4.1.1. Macroscopic Morphology of the Rust Layer

**Figure 1:**
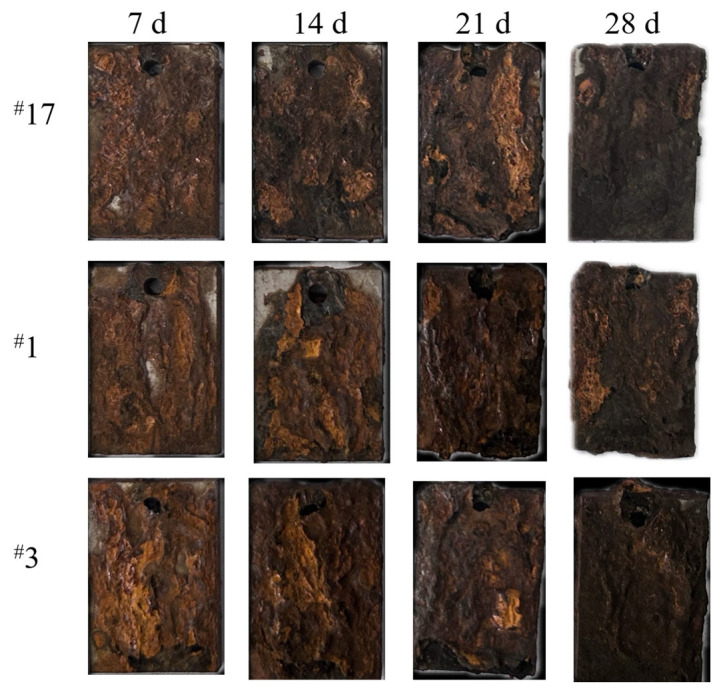
Macroscopic morphology of the experimental steel after corrosion.

The macroscopic corrosion morphology of the experimental steels over different corrosion periods is shown in Figure 1. In the early stage of corrosion (7 days), the rust layer was thin, brownish yellow in color, with a loose outer layer that easily flaked off. In the middle stage of corrosion (14 days), the rust layer appeared yellow–brown, with a denser outer layer and only slight detachment. As the corrosion period progressed, the rust layer deepened in color, and by the late stage of corrosion (28 days), the rust layer became smooth and chocolate-colored, adhering more tightly to the steel substrate.

Based on the changes in rust color and the ease of rust removal, it was inferred that the initial corrosion products were free iron ions, which gradually transformed into ferrous oxide, ferrous hydroxide, and iron hydroxides. As corrosion deepened, more stable corrosion products, such as Fe_3_O_4_ and α-FeOOH, formed [15], effectively reducing the corrosion rate of the experimental steels.

#### 4.1.2. Macroscopic Surface Morphology

**Figure 2:**
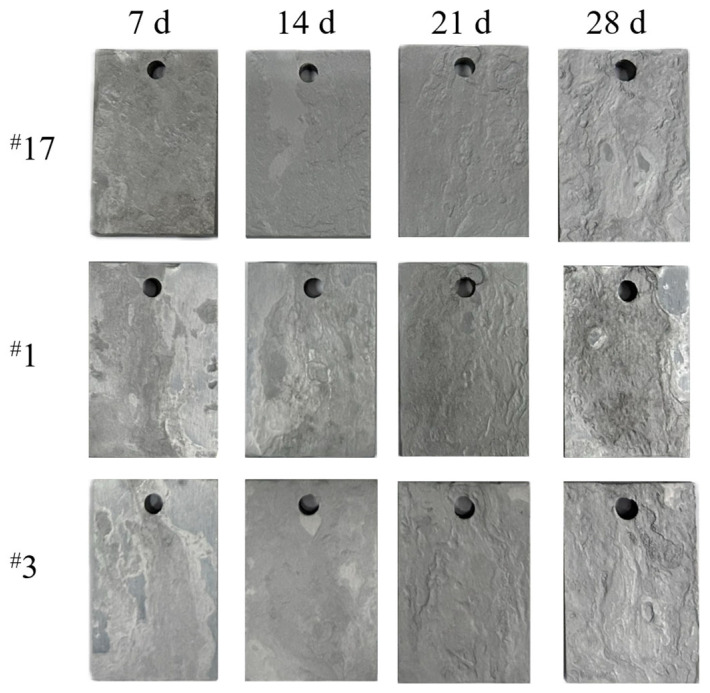
Macroscopic morphology of experimental steel after corrosion.

Figure 2 shows the macroscopic morphology after rust removal over different corrosion periods. In the first two periods, some areas of the surface remained smooth after rust removal. As the corrosion period lengthened, surface textures became more pronounced, and by the fourth period, noticeable corrosion blisters and river-like patterns appeared.

#### 4.1.3. Microscopic Surface Morphology

**Figure 3:**
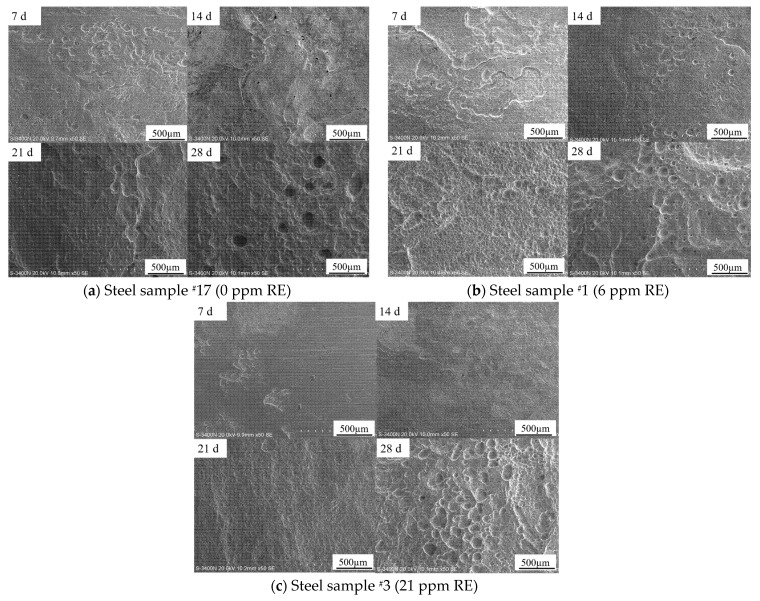
Microscopic surface morphology of the three groups of experimental steels after rust removal at different corrosion periods, observed by scanning electron microscopy.

Figure 3 presents magnified views of the surfaces after rust removal. In the early stages of corrosion, wave-like corrosion pits appeared on the surface of sample ^#^17, while the small pits on sample ^#^1 gradually merged into shallow depressions, and the surface of sample ^#^3 remained the flattest. During the corrosion process, small pits initially formed, leading to localized corrosion, which then increased and merged, eventually forming larger corrosion pits. The corrosion transitioned from localized in the early stages to uniform corrosion in the mid-stages. In the later stages of corrosion, deeper pitting appeared on the substrate surface, and a “scab” structure formed from a combination of the rust layer and corrosion pits, which further accelerated localized corrosion. The formation of the “scab” structure led to even deeper corrosion pits [16].

**Figure 4:**
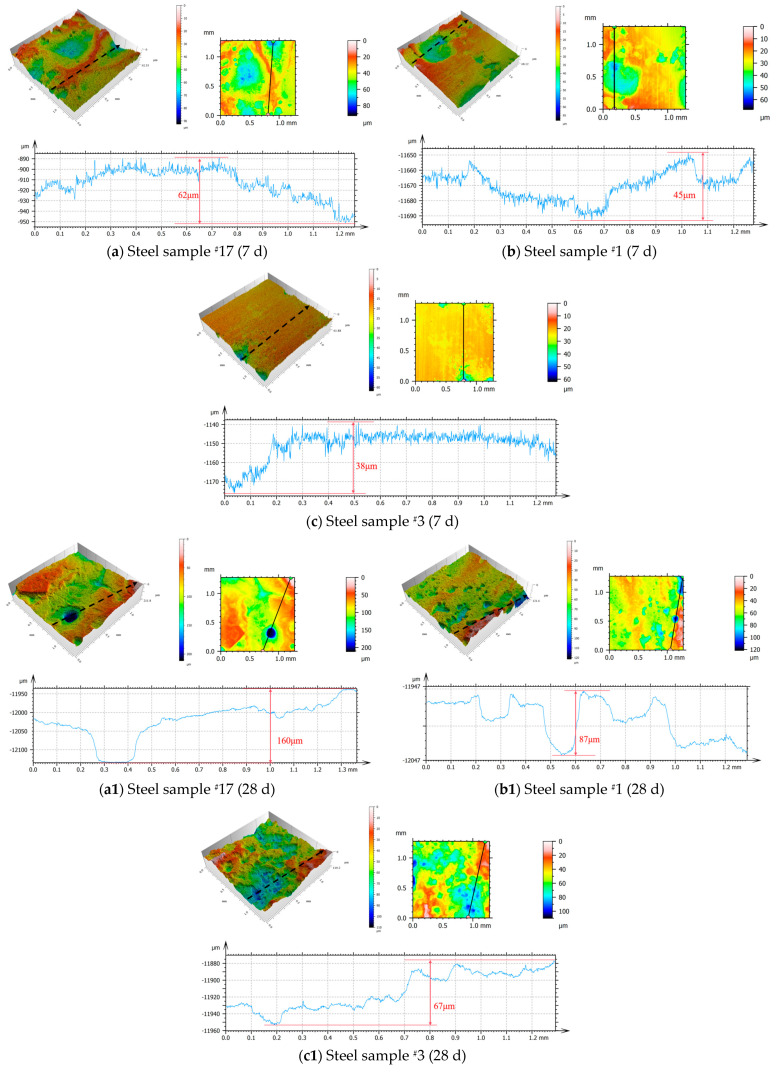
Microscopic surface morphology of the three groups of experimental steels after rust removal over different corrosion periods, observed by laser confocal microscopy.

Figure 4 shows the microscopic surface morphology observed by laser confocal microscopy, which is consistent with the scanning electron microscopy results. In the early stages of corrosion, the depths of the corrosion pits for samples ^#^17, ^#^1, and ^#^3 were 62 μm, 45 μm, and 38 μm, respectively, with sample ^#^3 exhibiting the smoothest surface and the least curve fluctuation. As the corrosion time increased, the depth of the pits continued to grow during the fourth period. Combined with Figure 3, it is evident that corrosion transitioned from localized corrosion in the early stages to more uniform corrosion. By the fourth period, localized corrosion intensified again, with sample ^#^17 experiencing the most severe localized corrosion, showing a height difference of up to 160 μm. Sample ^#^1 displayed numerous pitting pits with significant curve fluctuations, while sample ^#^3 exhibited extensive pit merging, resulting in a primarily localized, uniform corrosion pattern.

#### 4.1.4. Rust Layer Cross-Section Analysis

**Figure 5:**
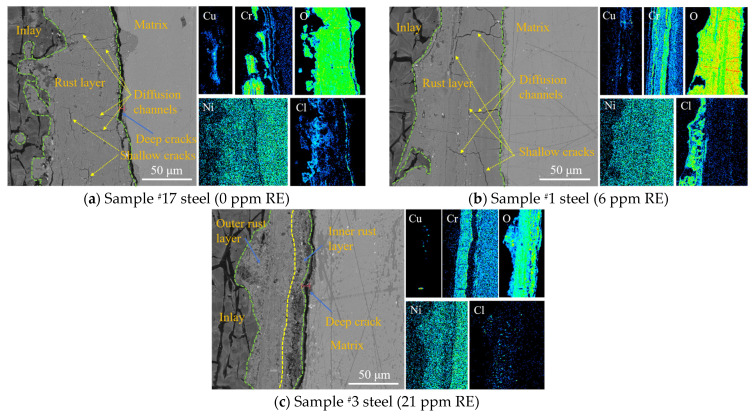
Elemental distribution in the rust layer cross-sections of the three experimental steels during the fourth corrosion cycle.

The cross-sections of the rust layers after 28 days of the salt spray test were analyzed using SEM-EDS, and the results are shown in Figure 5. In all three experimental steels, the Cr element was enriched in the rust layer near the substrate. Cr can substitute Fe in α-FeOOH, promoting the formation of amorphous hydroxides, such as α-(Fe_1−x_Cr_x_)OOH, which improves the rust layer structure. Additionally, the combined action of Cr and Cu forms a dense oxide layer that passivates the surface, preventing oxygen penetration and enhancing the rust layer’s density [17]. Compared to the steel without rare earth elements, the experimental steels with added Ce showed a more uniform distribution of Cr in the rust layer.

In sample ^#^17, the Cu element was mainly concentrated in the middle of the rust layer, whereas in samples ^#^1 and ^#^3, the Cu distribution was more dispersed, with sample ^#^3 having a lower Cu content. The presence of Cu can refine the rust layer’s grain structure, thereby enhancing its electrochemical protection performance [18]. In sample ^#^17, the Cu element was primarily concentrated in the middle of the rust layer, while in samples ^#^1 and ^#^3, the Cu distribution was more dispersed, with a lower Cu content observed in sample ^#^3. The Cu element can refine the grain structure of the rust layer, thereby enhancing its electrochemical protective performance [19]. The Ni element can shorten the transformation time from γ-FeOOH to α-FeOOH, catalyzing and refining α-FeOOH while also enhancing adhesion of the rust layer [20]. However, in the three groups of steel tested in this experiment, no significant enrichment of Ni was observed. The corrosion products contained a large amount of oxygen, and the rust layer was mainly composed of iron oxides, which is consistent with the phase analysis results. The Cl element in the rust layer primarily originated from marine salt particles in the atmosphere.

It is evident that, in the rust layer of experimental steel ^#^1, which contains rare earth elements, Cl ions are primarily distributed in the outer rust layer. In experimental steel ^#^3, no significant Cl enrichment is observed. Conversely, in the rust layer of experimental steel ^#^17, which lacks rare earth elements, Cl ions are more densely distributed, with Cl ions also segregating to the interface between the inner rust layer and the matrix. This is attributed to the numerous transverse cracks in the rust layer of steel ^#^17, which allow Cl^−^ to diffuse more easily to the interface with the matrix. This means that Cl ions can directly contact the steel matrix through the inner rust layer. Cl^−^ is one of the main factors contributing to steel corrosion, as it can initiate pitting, particularly at local defects or cracks, leading to the formation of small cavities that accelerate corrosion. Furthermore, the presence of Cl^−^ not only interferes with the formation of passive films but also reacts with them to form soluble chlorides, compromising the integrity of the passive film. This degradation promotes more intense electrochemical reactions on the local metal surface, resulting in localized corrosion, such as pitting, and accelerating the overall corrosion process. Compared to the experimental steel with rare earth elements, the steel without rare earth elements exhibits larger gaps at the rust layer interface, increasing the likelihood of Cl^−^ ions diffusing from these gaps to the matrix, thereby promoting corrosion. The rust layer of the experimental steel containing rare earth elements demonstrated better resistance to the ingress of Cl ions into the steel matrix. To some extent, the addition of the rare earth element Ce can effectively mitigate corrosion and reduce the corrosion rate.

### 4.2. Corrosion Rate

**Figure 6:**
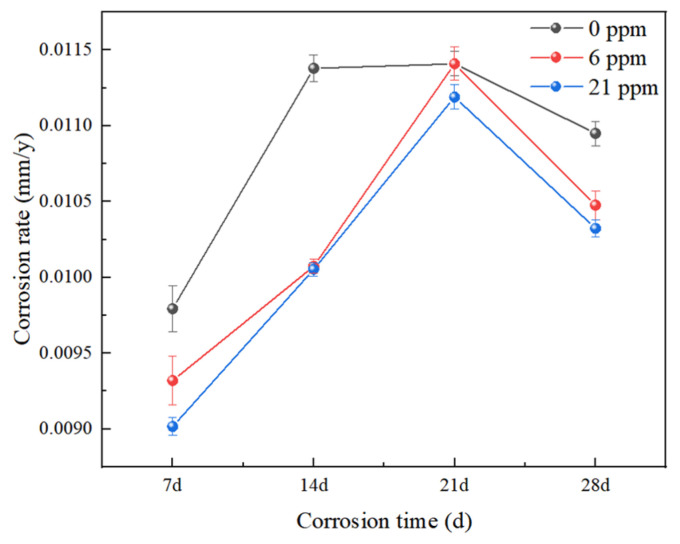
Corrosion rates of the three experimental steels at different corrosion periods.

As shown in Figure 6, the corrosion rate of all three groups of experimental steels showed an increasing trend before 21 days. At 14 days, the corrosion rate of sample ^#^17 was approximately 1.13 times that of samples ^#^1 and ^#^3. By 21 days, the corrosion rates of the three steels reached their peak, after which they began to decrease. The corrosion rate of the rare earth-containing steels decreased more significantly than that of the non-rare-earth steel. The non-rare-earth steel exhibited the fastest rate of increase and the slowest rate of decrease, maintaining a higher corrosion rate than the rare earth-containing steels throughout. This indicates that the addition of trace amounts of rare earth elements effectively improved the corrosion resistance of the experimental steels. Especially in the later stages of corrosion, the decrease in corrosion rate became more pronounced, indicating the effectiveness of Ce in corrosion protection. However, only a small amount of rare earth Ce needs to be added to steel. Excessive Ce concentration can lead to technical issues during production, such as nozzle clogging, thereby increasing production difficulty.

### 4.3. Phase Analysis

**Figure 7:**
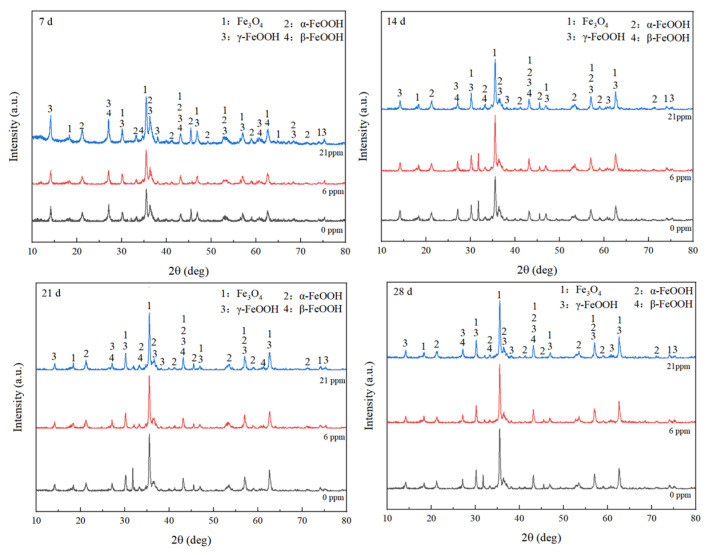
XRD spectra of the three experimental steels at different corrosion periods.

By analyzing the corrosion products of the samples over different corrosion periods, it was found that the composition of the corrosion product film evolved, eventually forming stable corrosion products. As shown in Figure 7, after four corrosion cycles, the phase composition of the corrosion products in the three groups of experimental steels was generally similar. The main product was magnetic Fe_3_O_4_, accompanied by stable α-FeOOH and active γ-FeOOH and β-FeOOH. In corrosion products, α-FeOOH, β-FeOOH, and γ-FeOOH are common iron oxyhydroxide phases. α-FeOOH is a stable crystalline phase with a fibrous structure, typically formed in environments with low redox potential and neutral to mildly alkaline pH. Its formation contributes to enhancing the density of the rust layer. Due to its dense and stable structure, α-FeOOH effectively prevents oxygen and moisture from further diffusing into the base metal, thus playing a critical role in protective rust layers. β-FeOOH, on the other hand, is considered a more aggressive corrosion product, often forming in chloride-rich environments, such as marine atmospheres. Its crystalline structure has narrow channels that can absorb chloride ions, making it relatively unstable. The presence of β-FeOOH results in a less dense rust layer and increases the likelihood of localized corrosion (e.g., pitting). This type of oxide typically has a negative impact on the steel’s corrosion resistance. γ-FeOOH is another common product generated during iron corrosion, characterized by a flaky structure. It usually forms in the initial stages of corrosion and exhibits relatively high activity. γ-FeOOH is comparatively less stable, and its layered structure creates more porosity, which can allow moisture and oxygen to penetrate the base metal, thus weakening the rust layer’s protective capability. However, under specific conditions, γ-FeOOH can gradually transform into α-FeOOH, enhancing the protective effectiveness of the rust layer [15,21,22]. In terms of corrosion products, the addition of rare earth elements does not have a significant impact on the phase composition of the rust layer. Typically, the intensity of XRD peaks correlates positively with the mass fraction of the corresponding substance [23]. It was observed that during the 14-day, 21-day, and 28-day corrosion periods, the diffraction peak intensity of Fe_3_O_4_ in the experimental steels was significantly higher than that of the 7-day period. Although Fe_3_O_4_ is a good conductor, it is generally considered protective due to its density and thermodynamic stability [24].

**Figure 8:**
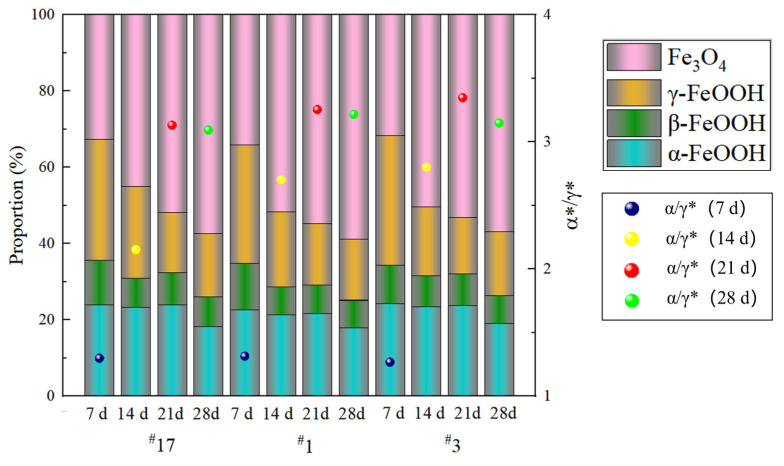
Semi-quantitative analysis of rust layers of different cycles for three groups of experimental steel.

To more accurately investigate the influence of rare earth elements on the corrosion products of the experimental steels, the relative intensity ratio (RIR) method was used for semi-quantitative phase analysis of the corrosion products [25], as shown in Figure 8. During the corrosion process, γ-FeOOH initially formed on the steel surface due to its high reactivity. As the alloying elements in the steel interacted with the alternating wet and dry atmospheric conditions, the rust layer gradually thickened, and γ-FeOOH was reduced to the less reactive Fe_3_O_4_ and the thermodynamically stable α-FeOOH [21,22,26,27]. Although both γ-FeOOH and β-FeOOH are considered reductive phases [28], β-FeOOH has the most detrimental effect on the corrosion resistance of the rust layer among all iron hydroxides [29]. β-FeOOH typically forms in high Cl^−^ environments, as Cl^−^ can stabilize its crystal tunnel structure [28,30]. Some researchers believe that Cl^−^ plays a catalytic role in the formation of β-FeOOH [31]. On the other hand, α-FeOOH and amorphous components help improve the protective properties of the rust layer [32,33,34], with α-FeOOH, as a stable phase, effectively inhibiting Cl^−^ penetration [35].

The PAI (protection ability index) was initially proposed by Yamashita et al. [15] as an index to evaluate the protective capacity of rust layers, defined as the ratio of needle-like goethite to fibrous goethite (α/γ). A higher PAI value indicates a more stable corrosion system. As the complexity of corrosion products increases, researchers have revised the significance and calculation formula of the α/γ ratio for different corrosion environments. Dillmann et al. [36] found that the protective capacity of the rust layer is positively correlated with the ratio of (α-FeOOH + Fe_3_O_4_) to γ-FeOOH. Liu Wei et al. [37] summarized the PAI formulas for various types of steel in different environments. The protective capacity of the rust layer for the three experimental steels was evaluated using the following formula (1), with results shown in Table 2.

(1) $$\mathrm{PAI}(\alpha */\gamma *)=\frac{\alpha -\mathrm{FeOOH}+{\mathrm{Fe}}_{3}O_{4}}{\beta -\mathrm{FeOOH}+\gamma -\mathrm{FeOOH}\;}$$

The test results indicate that, as the corrosion time increases, the PAI value gradually rises, reaching its maximum in the third cycle, at which point the protective ability of the rust layer is optimal. This finding aligns with the weight loss tests, which showed a downward trend in corrosion rates from the third to the fourth cycle, demonstrating that the experimental steel with added rare earth elements possesses superior rust layer protection capabilities.

### 4.4. Electrochemical Analysis

**Figure 9:**
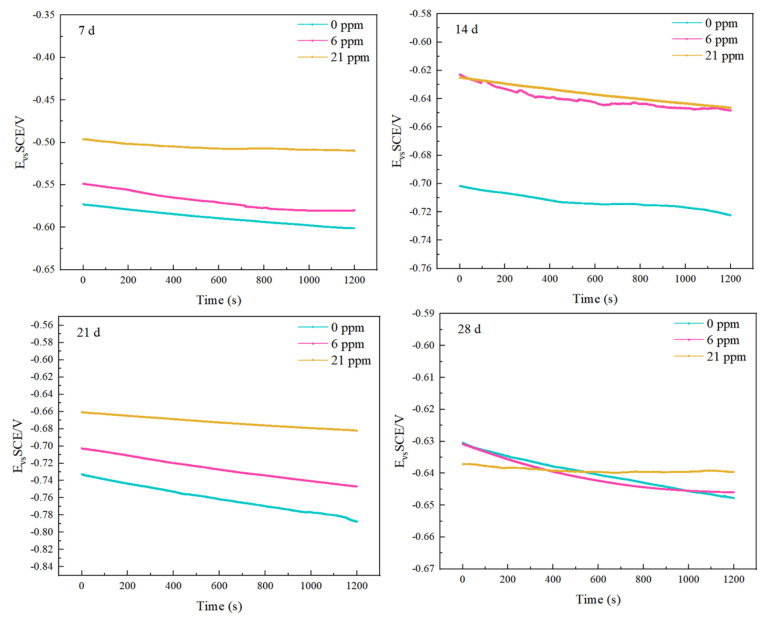
Open circuit voltages of the three groups of experimental steels over different cycles.

The open circuit potentials of the three groups of experimental steels were tested at different corrosion cycles, as shown in Figure 9. When the corrosion times were 7 days, 14 days, and 21 days, the open circuit voltages decreased in the order of experimental steels with 21 ppm, 6 ppm, and those without added rare earth elements. As the testing duration increased, the open circuit potentials gradually stabilized, indicating that the experimental system reached a steady state. In the fourth phase of the corrosion cycle, all three groups of experimental steels exhibited a rising trend in open circuit voltage, with the open circuit potential curve of sample ^#^3 being the most stable.

**Figure 10:**
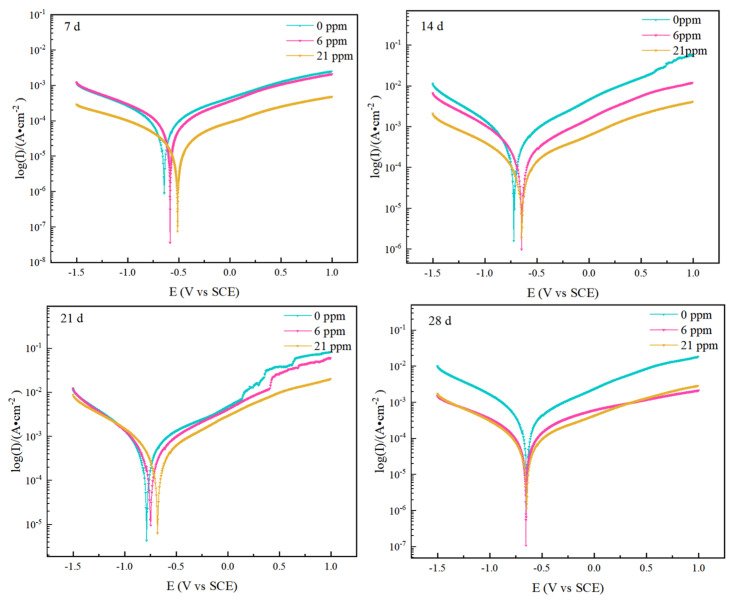
Polarization curves of the three groups of experimental steels over different cycles.

Figure 10 shows the dynamic polarization curves of rusted samples after different corrosion cycles. The curves appear relatively smooth, with no significant passivation observed during the four cycles of the salt spray test. As the corrosion time increased, the corrosion potential of the three groups of experimental steels shifted negatively, the corrosion current density increased, and the corrosion rate accelerated. This is mainly due to the loose and unstable rust layer at this stage, which cannot effectively protect the substrate. The corrosion process is primarily controlled by the limited diffusion of dissolved oxygen and the reduction of corrosion products. The corrosion rate peaked at 21 days, consistent with the weight loss test results. By 28 days, the polarization curves shifted to the right, with an increase in corrosion potential and a decrease in corrosion current density, indicating a slowdown in the corrosion rate. This suggests that a more stable corrosion product was formed at this stage, inhibiting the anodic dissolution of the steel and slowing the corrosion of the substrate. In the first three cycles, the experimental steels with added rare earth elements exhibited higher corrosion potentials at the same current density, effectively mitigating the corrosion rate. By the fourth cycle, the corrosion process had essentially stabilized.

**Figure 11:**
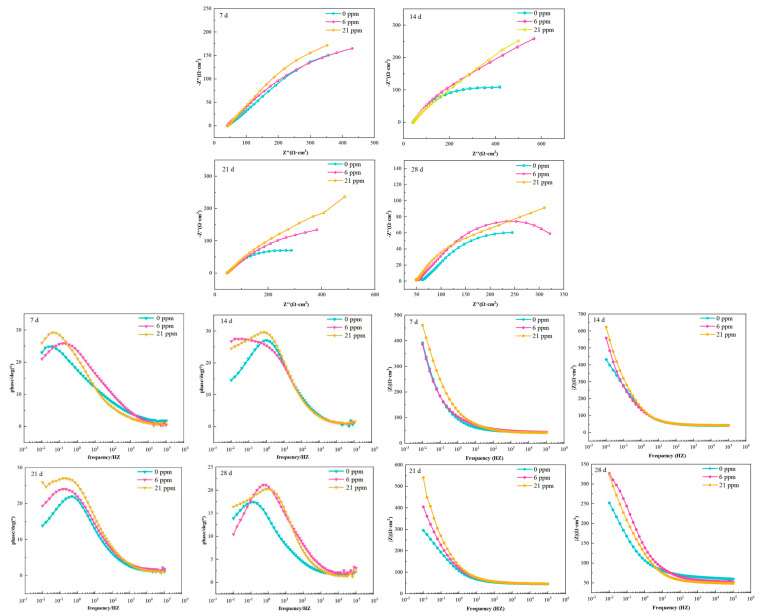
Electrochemical impedance spectra of the three groups of experimental steels over different cycles.

Figure 11 shows the impedance spectra of the three types of experimental steels during the salt spray test. In different test cycles, the shape of the capacitive arcs in the high-frequency region is irregular primarily due to the accumulation of corrosion products and the uneven or rough surface, which affects the charge and discharge processes on the electrode surface [38,39]. In the first two corrosion cycles, the Nyquist plots of the three samples show only one capacitive arc; however, in the third and fourth cycles, the Nyquist plots of sample ^#^3 consist of an incomplete semicircle in the high-frequency region and a diffusion tail in the low-frequency region. This is because, as corrosion time increases, the rust layer thickens, covering the steel surface entirely, obstructing mass transfer, and limiting the transport of O_2_ and Cl^−^ to the substrate, making diffusion control the dominant factor [40,41]. Additionally, the experimental steels with added rare earth elements show a larger high-frequency semicircle radius throughout the salt spray test. The diameter of the capacitive arc in the Nyquist plot is typically associated with the corrosion resistance of the passivation film on the surface of the steel or alloy [42], with a larger capacitive arc diameter indicating stronger corrosion resistance of the passivation film, suggesting that an appropriate amount of rare earth elements helps form a more protective rust layer on the experimental steel. In the Bode plots, the low-frequency peak of the phase angle corresponds to the low-frequency arc in the Nyquist plot. A higher phase angle value indicates a more stable passivation film. During the salt spray test, the passivation film maintains a high phase angle in the mid-to-low-frequency region across different cycles, indicating that a stable passivation film formed on the steel surface. Similarly, the impedance modulus in the low-frequency region remains high throughout the four corrosion cycles, with the experimental steels containing rare earth elements exhibiting better stability.

### 4.5. Calculation of Adhesion Force Between the Rust Layer and Substrate

**Figure 12:**
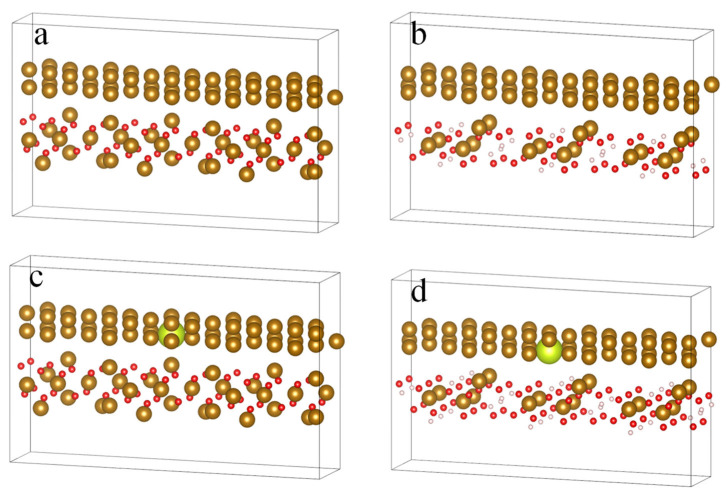
Interface structure between different rust layers and the substrate. (**a**) Fe-Fe_3_O_4_, (**b**) Fe-α-FeOOH, (**c**) FeCe-Fe_3_O_4_, and (**d**) FeCe-α-FeOOH.

In first-principles calculations, the work of adhesion is a physical quantity used to describe the adhesion energy between different materials. The work of adhesion at the interface between the substrate and the rust layer represents the strength or adhesion energy between these two different materials [43,44]. During the metal corrosion process, an interface exists between the metal substrate and the rust layer it forms. The larger the value of the work of adhesion at this interface, the stronger the adhesion between the substrate and the rust layer, leading to a tighter bond. Therefore, the magnitude of the adhesion work directly influences the stability of the interface between the metal substrate and the rust layer [45,46,47,48]. A larger value of the interface adhesion work indicates a tighter bond between the metal substrate and the rust layer, resulting in a more stable interface that can effectively resist further corrosion or oxidation from the external environment. Conversely, a lower value of adhesion work may lead to a loose interface that is prone to peeling, making the metal substrate more susceptible to corrosion or oxidation. The interface structure between the substrate and the rust layer is shown in Figure 12. In this context, the brownish-yellow spheres, green spheres, red spheres, and white spheres represent the Fe, Ce, O, and H elements, respectively. 

The calculation formula is written as [47,48]:

(2) $$W_{\mathrm{ad}}=(E_{\mathrm{matrix}}^{\mathrm{slab}}+E_{\mathrm{rust}\;\mathrm{layer}}^{\mathrm{slab}}-{\;E}_{m/r}^{\mathrm{interface}})/A$$

where: 

$E_{\mathrm{matrix}}^{\mathrm{slab}}$—the energy of the substrate (eV);

$E_{\mathrm{rust}\;\mathrm{layer}}^{\mathrm{slab}}$—the energy of the rust layer (eV);

${\;E}_{m/r}^{\mathrm{interface}}$—the total energy of the substrate/rust layer model (eV);

*A*—the interface area (Å^2^).

Table 3 shows that, by calculating the stability between the crystal structures of several common inner rust layers and the substrate, the adhesion work of these substrate/rust layer interfaces is all greater than 0, indicating that their interfaces are theoretically stable. The adhesion work between FeCe-α-FeOOH and Fe-α-FeOOH, as well as between FeCe-Fe_3_O_4_ and Fe-Fe_3_O_4_, shows significant differences. This demonstrates that the appropriate addition of rare earth elements can enhance the adhesion work between the iron substrate and the rust layer, thereby improving their bonding strength and stability.

The addition of rare earth elements can alter the lattice structure, surface morphology, and electronic structure of the metal substrate, thereby forming stronger interactions with the rust layer. This enhanced interface bonding helps prevent the penetration of corrosive substances from the external environment, slowing down the corrosion rate of the metal and thus improving the material’s durability. Furthermore, the increase in the work of adhesion after adding rare earth elements may suggest that their introduction has changed the chemical properties of the metal surface. Rare earth elements have unique chemical reactivity and oxidation characteristics, allowing them to form chemical or ionic bonds with oxygen atoms in the rust layer. The formation of these chemical bonds strengthens the mutual adsorption and interaction between the metal substrate and the rust layer, thereby improving the stability and corrosion resistance of the interface.

## 5. Conclusions

(1) In a simulated marine atmospheric corrosion environment, the corrosion rate of the experimental steel without added rare earth Ce was higher than that of the steel with added Ce across all four test cycles, with the corrosion rate on day 14 being approximately 1.13 times that of the rare earth-added steel. The steel with added rare earth exhibited better atmospheric corrosion resistance. In the salt spray test, the upper limit values of α*/γ* appeared in the third cycle, which is one of the reasons for the decline in corrosion rate after the third cycle.
(2) The rust layer of the experimental steel can be divided into an inner rust layer and an outer rust layer. The addition of cerium significantly promotes the formation of a protective rust layer. In the rare earth-added steel, Cr elements were mainly concentrated in the inner rust layer, whereas Cl elements in the rust layer of steel ^#^1 were mainly distributed in the outer rust layer. No significant accumulation of Cl elements was observed in the rust layer of steel ^#^3, while the rust layer of the non-rare earth-added steel ^#^17 showed obvious segregation of Cl elements. This made Cl elements more likely to penetrate the inner rust layer and reach the steel substrate, thereby accelerating corrosion. Additionally, no excessively wide cracks were found in the rust layer of steel ^#^1. Although a relatively wide crack measuring 5.223 μm was observed between the substrate and rust layer of steel ^#^3—slightly smaller than the 6.126 μm crack in steel ^#^17—the good compactness of the rust layer in steel ^#^3 still effectively hindered the corrosion process. Ce can promote the enrichment of Cr in the inner rust layer and form a denser protective layer, which hinders the penetration of oxygen ions, helping to slow down the corrosion process.
(3) The polarization curves of the three groups of experimental steels were relatively smooth, with no passivation observed. As the corrosion time increased, the corrosion potentials of the three steels shifted negatively, and the corrosion current density increased, indicating a faster corrosion rate at this stage. As corrosion progressed, after the corrosion current reached its maximum in the third cycle, the polarization curves shifted to the right, with a rising trend in corrosion potential and a decrease in corrosion current density, slowing the corrosion process during this stage.
(4) Calculation results show that the addition of rare earth Ce increases the adhesion work between the steel substrate and the rust layer by 0.141 J/m^2^ and 0.103 J/m^2^, respectively. The bonding strength between the test steel with added rare earth Ce and the rust layer is greater than that of the test steel without the rare earth addition. The addition of rare earth enhanced the adhesion and compactness of the rust layer, thereby improving the corrosion resistance of the experimental steels.

## Figures and Tables

**Table 1:** The three test steel chemistries.

Sample	C	Si	Mn	P	S	Al	Cr	Cu	Nb	Ni	Ce
^#^17	0.055	0.17	1.12	0.010	<0.002	0.032	0.53	0.31	0.043	0.31	
^#^1	0.067	0.16	1.10	0.011	<0.002	0.020	0.55	0.35	0.034	0.32	0.0006
^#^3	0.065	0.15	1.09	0.011	<0.002	0.030	0.55	0.34	0.036	0.32	0.0021

**Table 2:** Ratio of the rust layer protection ability of the test steel with different rare earth contents after the neutral salt spray test.

	Time	7 d	14 d	21 d	28 d
Sample					
^#^17 (0 ppm)		1.299	2.155	3.132	3.094
^#^1 (6 ppm)		1.315	2.704	3.255	3.218
^#^3 (21 ppm)		1.268	2.802	3.348	3.149

**Table 3:** Adhesion work between different rust layers and substrate.

Interface Structure	E_Fe_/eV	E_rust_/eV	E_total_/eV	a/Å	b/Å	W_ad_/j/m^2^
Fe-α-FeOOH	−325.413	−478.992	−813.637	6.1592	30.7962	0.780
FeCe-α-FeOOH	−325.267	−478.993	−815.161	6.1592	30.7962	0.921
Fe-Fe_3_O_4_	−325.406	−483.274	−816.487	6.1592	30.7962	0.659
FeCe-Fe_3_O_4_	−325.267	−483.274	−817.568	6.1592	30.7962	0.762

## Data Availability

The original contributions presented in the study are included in the article, further inquiries can be directed to the corresponding author.
